# The Global Immune–Nutrition–Inflammation Index (GINI) as a Candidate Prognostic Marker in Patients with Cutaneous Squamous Cell Carcinoma Treated with Cemiplimab

**DOI:** 10.3390/cancers18172862

**Published:** 2026-09-04

**Authors:** Tara Coreanu, Ido Amir, Nofar Edri, Itamar Averbuch, Aviram Mizrachi, Amit Ritter, Moran Amit, Noga Kurman, Eyal Yosefof, Dan Yaniv

**Affiliations:** 1 Department of Otorhinolaryngology and Head and Neck Surgery, Rabin Medical Center, Beilinson Hospital, Petah Tikva 4941492, Israel; 2 Gray Faculty of Medicine and Health Sciences, Tel Aviv University, Tel Aviv 6997801, Israel; 3 Davidoff Cancer Center, Rabin Medical Center, Petah Tikva 4941492, Israel; 4 Department of Otolaryngology-Head and Neck Surgery, Weill Cornell Medical College, Cornell University, New York, NY 10022, USA; 5 Department of Head and Neck Surgery, The University of Texas MD Anderson Cancer Center, Houston, TX 77030, USA

**Keywords:** GINI, cSCC, Cemiplimab

## Abstract

**Background/Objectives**: Cutaneous squamous cell carcinoma (cSCC) is a highly prevalent malignancy. While Cemiplimab has transformed the treatment landscape across various disease stages, reliable pretreatment biomarkers for risk stratification remain lacking.. The Global Immune–Nutrition–Inflammation Index (GINI) is a composite biomarker integrating systemic inflammation and nutritional status into a single score. This study aimed to evaluate the prognostic value of the pretreatment GINI in patients with cSCC receiving Cemiplimab across all treatment settings. **Methods**: This retrospective cohort study evaluated 73 patients with unresectable, locally advanced, or metastatic cSCC treated with Cemiplimab between 2020 and 2023. Pretreatment laboratory parameters were extracted to calculate the GINI score. Receiver operating characteristic (ROC) curve analysis determined the optimal GINI cutoff to stratify patients into low- and high-GINI groups. Survival outcomes, including overall survival (OS) and progression-free survival (PFS), were analyzed using Kaplan–Meier curves and multivariable Cox proportional hazards models. **Results**: An optimal GINI cutoff of 74.4 divided the cohort into low-GINI (38%) and high-GINI (62%) groups. In the multivariable Cox regression models, a high pretreatment GINI remained independently associated with both inferior OS (adjusted HR 2.51, 95% CI 1.05–6.01, *p* = 0.039) and inferior PFS (adjusted HR 3.22, 95% CI 1.45–7.14, *p* = 0.004). When compared against five established inflammatory biomarkers (NLR, PLR, LMR, PNI, and CAR), the GINI consistently demonstrated comparable prognostic performance across multiple statistical approaches. **Conclusions**: The pretreatment GINI is a candidate prognostic marker for patients with cSCC undergoing Cemiplimab therapy. Given its cost-effectiveness and ready availability from routine clinical laboratory workups, the GINI score may improve prognostic risk stratification and could potentially inform risk assessment and clinical monitoring in real-world oncological practice.

## Simple Summary

Cutaneous squamous cell carcinoma (cSCC) is the second most common type of skin cancer. Although anti-PD-1 immunotherapy has significantly improved treatment outcomes for patients with advanced disease, a substantial portion of individuals do not respond, and reliable pretreatment tools to predict success are currently lacking. This study explores whether a scoring system called the Global Immune–Nutrition–Inflammation Index (GINI), which combines routine laboratory measurements of inflammation and nutritional status, can predict survival in cSCC. The findings demonstrate that a high GINI score is strongly linked to poorer treatment response and shorter survival. This practical index has the potential to help clinicians identify high-risk patients early and inform risk assessment and clinical monitoring, potentially supporting future treatment planning.

## 1. Introduction

Cutaneous squamous cell carcinoma (cSCC) is the second most common malignancy in humans, with incidence rates having increased by more than 260% over recent decades and an estimated annual burden exceeding 700,000 new cases in the United States alone [1,2]. Although most cases are cured by surgery or radiotherapy at the localized stage, approximately 3–5% of patients develop locally advanced or metastatic disease, which carries a poor prognosis and, prior to 2018, had no approved systemic therapy [3]. The introduction of Cemiplimab, a fully human anti-PD-1 monoclonal antibody, transformed this landscape across all disease stages. In the pivotal phase 2 EMPOWER-CSCC-1 trial, Cemiplimab achieved objective response rates of approximately 47% in metastatic and locally advanced cSCC, with durable responses maintained in over 87% of responders at 12 months [4,5]. In the neoadjuvant setting, Gross et al. demonstrated pathological complete response in 51% of patients with resectable stage II–IV cSCC [6], and in the phase 3 C-POST trial, adjuvant Cemiplimab reduced the risk of recurrence by 68% compared to placebo in high-risk patients following surgery and radiotherapy [7]. Despite these advances, a substantial proportion of patients do not respond to Cemiplimab regardless of disease stage, and reliable pretreatment biomarkers to inform risk assessment remain lacking.

The Global Immune–Nutrition–Inflammation Index (GINI), calculated as (C-reactive protein × monocytes × platelets × neutrophils)/(albumin × lymphocytes), is a composite blood-based biomarker that integrates systemic inflammation and nutritional status into a single score. Across multiple solid tumor types, a high GINI has been consistently associated with inferior oncologic outcomes. While Topkan et al. [8] first introduced the GINI, Aoyama et al. first validated it in 258 gastric cancer patients undergoing curative resection, identifying a high GINI as an independent predictor of overall survival (HR 1.77) [9]. In esophageal squamous cell carcinoma, the GINI predicted pathological response to neoadjuvant immunochemotherapy with an AUC of 0.912 and independently predicted survival [10]. In stage IIIC non-small cell lung cancer, a high GINI was associated with a halving of median overall survival (19.1 vs. 37.8 months) [8], and in metastatic colorectal cancer, a more than threefold increase in the OS hazard ratio with a four-year survival differential [11]. In glioblastoma, the GINI outperformed established inflammatory indices including the systemic immune–inflammation index and pan-immune–inflammation value as an independent prognostic factor [12]. No study to date has evaluated the GINI in cSCC.

Numerous inflammatory biomarkers, including the neutrophil-to-lymphocyte ratio (NLR) [13], platelet-to-lymphocyte ratio (PLR) [14], lymphocyte-to-monocyte ratio (LMR) [15], prognostic nutritional index (PNI) [16], and C-reactive protein-to-albumin ratio (CAR) [17], have been proposed as prognostic indicators in oncology. However, comparative data evaluating these indices specifically in patients receiving immunotherapy for cutaneous SCC remain limited. Therefore, we performed an exploratory comparison of GINI with established inflammatory biomarkers.

The aim of this study was to evaluate the prognostic value of the pretreatment GINI in patients with cSCC receiving Cemiplimab across all treatment settings. We hypothesized that a high pretreatment GINI, reflecting the convergence of systemic inflammation and impaired nutritional and immune reserve, would be independently associated with inferior treatment outcomes in this population.

## 2. Materials and Methods

This retrospective cohort included all cSCC patients who were treated with Cemiplimab at a university-affiliated tertiary care center between 2020 and 2023. In order to maintain cohort homogeneity and ensure data completeness, patients treated with PD-1 inhibitors other than Cemiplimab or patients with incomplete data were excluded.

Clinical and demographic data was collected from patients’ electronic medical records (EMR), including Eastern Cooperative Oncology Group (ECOG) performance status [18], primary tumor location, TNM staging based on the 8th version of the American Joint Committee of Cancer (AJCC) guidelines [19] and prior treatment modalities.

Data regarding Cemiplimab treatment were collected, including the time of treatment initiation and termination, whether Cemiplimab was used as first-line therapy, number of treatment cycles, first response to treatment, time to first response, maximal response to treatment and time to maximal response, progression on treatment, and time to progression. Response to treatment was defined as either complete response (CR), partial response (PR), stable disease (SD), or progression of disease (PD). Tumor response was assessed based on documentation in the electronic medical records (EMRs), including radiology reports (one or more consecutive PET-CT scans) and the treating oncologist’s clinical assessment. Formal RECIST 1.1 criteria [20] were not applied. Instead, responses were categorized according to the treating physician’s interpretation of radiologic findings and clinical notes, reflecting real-world practice. Objective response rate (ORR) was defined as the rate of patients with either CR or PR out of all patients in the cohort, while disease-control rate (DCR) was defined as patients with either CR, PR, or SD. Best response was summarized among response-evaluable patients only. Response was evaluable in 62 of 73 patients overall (Low GINI: 26/28; High GINI: 36/45).

Treatment toxicity was assessed using the grading system suggested by the Common Terminology Criteria for Adverse Events (CTCAE) [21]. Data regarding specific adverse effects were also collected and categorized into immune-related adverse events (irAEs) and treatment-related adverse events (trAEs). Specifically, irAEs included diarrhea, hypothyroidism, hyperthyroidism, myocarditis, nephritis, hepatitis, type 1 diabetes mellitus, adrenal insufficiency, colitis, Guillain-Barré syndrome, myasthenia gravis, and the myasthenia–myositis–myocarditis overlap syndrome.

The primary outcomes of the study included overall survival (OS) and progression-free survival (PFS). The secondary outcome was the rate of treatment-related toxicities.

### Statistical Analysis

Continuous variables are presented as mean ± standard deviation (SD) and categorical variables as frequencies and percentages. Comparisons between low- and high-GINI groups were performed using the Wilcoxon rank-sum test for continuous variables and Fisher’s exact test for categorical variables.

The Global Immune–Nutrition–Inflammation Index (GINI) was calculated using the following formula:

GINI = (C-reactive protein × platelet count × monocyte count × neutrophil count)/(albumin × lymphocyte count)

In addition to GINI, the neutrophil-to-lymphocyte ratio (NLR), platelet-to-lymphocyte ratio (PLR), lymphocyte-to-monocyte ratio (LMR), prognostic nutritional index (PNI), and C-reactive protein-to-albumin ratio (CAR) were calculated from baseline laboratory values obtained before initiation of Cemiplimab therapy.

Receiver operating characteristic (ROC) curve analysis was performed to evaluate the ability of baseline GINI to predict overall survival (OS). The optimal GINI cutoff was determined using the Youden index and subsequently used to dichotomize patients into low- and high-GINI groups. Cutoff internal stability was assessed using 10,000 bootstrap resamples.

To compare the prognostic performance of inflammatory biomarkers, ROC analyses were additionally performed for NLR, PLR, LMR, PNI, and CAR. Optimal cutoffs for each biomarker were determined using the Youden index, and biomarkers were dichotomized according to their established biological direction (higher values indicating greater risk for GINI, NLR, PLR, and CAR; lower values indicating greater risk for PNI and LMR). Areas under the ROC curve (AUCs) with 95% confidence intervals were calculated, and ROC curves were compared using DeLong’s test for paired data.

Survival curves were estimated using the Kaplan–Meier method and compared using the log-rank test.

Associations between GINI group and treatment-related toxicity were evaluated using Fisher’s exact test. Two toxicity endpoints were assessed: (1) occurrence of any treatment-related toxicity and (2) occurrence of grade ≥ 3 toxicity. Logistic regression models were used to estimate odds ratios (ORs) with corresponding 95% confidence intervals (CIs). Individual recorded adverse events were also compared between GINI groups.

Univariable Cox proportional hazards regression analyses were performed for OS and progression-free survival (PFS). To limit overfitting given the cohort size and number of outcome events, the primary multivariable Cox models included GINI group, age at Cemiplimab initiation, and disease stage. Hazard ratios (HRs) with 95% confidence intervals (CIs) were reported.

For the biomarker comparison analyses, separate multivariable Cox models were fitted for each inflammatory biomarker, with each model including a single biomarker together with the same predefined clinical covariates to permit direct comparison of prognostic performance. Hazard ratios (HRs) with 95% confidence intervals (CIs), Harrell’s concordance index (C-index), Akaike information criterion (AIC), Bayesian information criterion (BIC), and log-likelihood were reported for each model.

The proportional hazards assumption was assessed using the Grambsch–Therneau test based on scaled Schoenfeld residuals. A time-varying model was calculated to account for proportional hazard violations when applicable.

Comparative biomarker analyses were restricted to patients with complete data for all inflammatory biomarkers and survival outcomes to ensure that all biomarkers were evaluated in an identical cohort. Of 133 patients treated with Cemiplimab, 73 had complete data and were included in the comparative survival analyses [Supplementary Figure S1].

All statistical tests were two-sided, and *p*-values < 0.05 were considered statistically significant. Statistical analyses were performed using R version 4.5.3 (R Foundation for Statistical Computing, Vienna, Austria).

## 3. Results

### 3.1. Patients

A total of 73 patients with unresectable, locally advanced, or metastatic cSCC who received Cemiplimab between 2020 and 2023 were evaluated. The optimal GINI cutoff was 74.4 (area under the curve: 0.659, sensitivity: 0.79, specificity: 0.52, Youden index: 0.313). Patients were stratified into a low-GINI group (n = 28, 38%) and a high-GINI group (n = 45, 62%). Baseline demographic and clinical characteristics were well-balanced between the two cohorts (Table 1). Males predominated in both the low- and high-GINI cohorts (82% vs. 80%, *p* > 0.9). The mean age at Cemiplimab initiation was nearly identical between the groups (78.84 ± 8.03 vs. 78.43 ± 13.16, *p* = 0.8), and no significant differences were observed regarding ECOG performance status (*p* = 0.5), with a substantial proportion of patients presenting with an ECOG score > 2 (43% vs. 44%, respectively).

Consistent with established epidemiological patterns, the head and neck was the most frequent primary tumor site in both arms (71% vs. 60%, *p* = 0.6). Tumor staging features were equally distributed across the groups; half of the patients presented with early-stage local disease (T1 or T2: 50% vs. 54%, *p* > 0.9) and the majority had a clinically negative nodal status (N0: 79% vs. 82%, *p* > 0.9). Prior definitive therapeutic interventions did not vary significantly, with surgical resection with or without adjuvant therapy comprising the primary prior treatment modality in the high-GINI group (50% vs. 63%, *p* = 0.6). Notably, the duration of Cemiplimab therapy was shorter in the high-GINI group (7.86 ± 7.17 months) compared to the low-GINI group (11.25 ± 8.18 months, *p* = 0.06).

As expected, notable differences were observed in baseline laboratory values between the cohorts: the high-GINI group demonstrated significantly lower albumin levels (3.73 ± 0.49 vs. 4.12 ± 0.32 g/dL, *p* < 0.001), significantly higher C-reactive protein levels (4.69 ± 5.23 vs. 0.45 ± 0.75, *p* < 0.001), higher neutrophil counts (7.18 ± 3.61 vs. 4.38 ± 1.58, *p* < 0.001), and higher platelet counts (256.71 ± 99.56 vs. 185.93 ± 71.06, *p* < 0.001). Lymphocyte (1.61 ± 1.48 vs. 2.05 ± 1.72, *p* = 0.10) and monocyte (0.77 ± 0.68 vs. 0.78 ± 0.66, *p* = 0.7) counts did not significantly differ.

### 3.2. Response to Treatment

The time to best response was slightly shorter in the high GINI score group (3.9, 2–7.9 vs. 5.95, 3.70–19.10 months, *p* = 0.3) as seen in Table 2. Regarding the depth of response, the objective response rate (ORR) was 85% (22/26 patients) in the low-GINI group compared to 70% (28/36 patients) in the high-GINI group. The disease control rate (DCR) was 88% (23/26 patients) for the low-GINI cohort and 86% (31/36 patients) for the high-GINI cohort. A complete response (CR) was achieved in 42% (n = 11) of patients with low GINI, whereas only 28% (n = 10) of patients in the high-GINI group reached CR. Partial responses (PR) were observed in 42% (n = 11) and 50% (n = 18) of patients, while progressive disease (PD) was documented in 12% (n = 3) and 14% (n = 5) of the low- and high-GINI groups, respectively. Stable disease (SD) was noted in 4% (n = 1) vs. 8% (n = 3) of patients. However, these distributions of response did not reach statistical significance (*p* = 0.7). There were higher rates of death in the high-GINI group compared with the low-GINI group (58% vs. 25%, *p*-value 0.008).

### 3.3. Survival Rates

**Figure 1:**
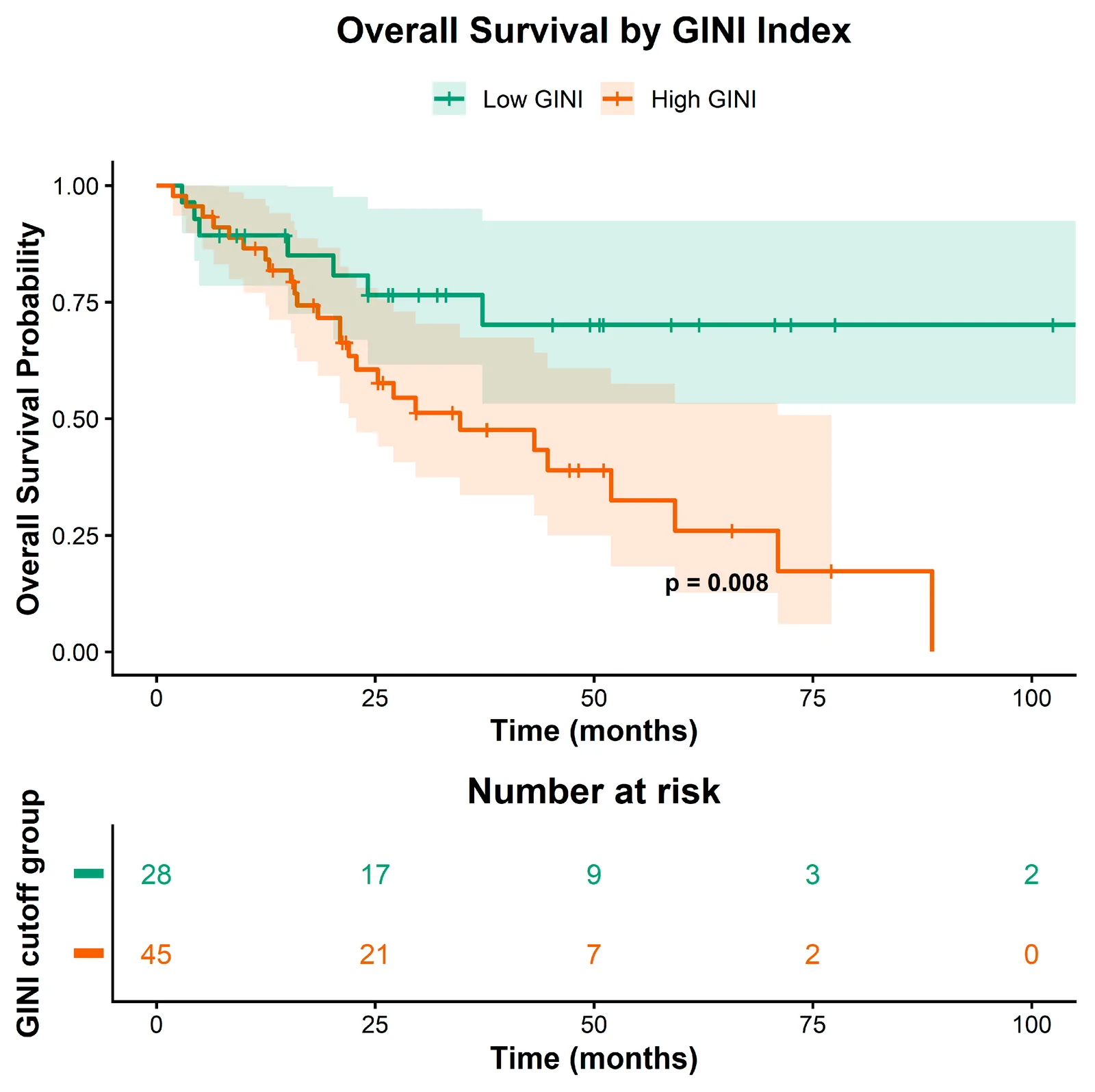
Overall survival KM by GINI.

In the multivariable Cox regression model for OS the high-GINI group remained independently associated with inferior overall survival (adjusted HR 2.51, 95% CI 1.05–6.01, *p* = 0.039) [Figure 1]. Advanced age was also independently associated with inferior OS (adjusted HR 1.08, 95% CI 1.04–1.12, *p* < 0.001). Female sex (*p* = 0.627), smoking status (*p* = 0.231), advanced disease stage (Stage III-IV; *p* = 0.691), and radiation-based prior treatment (*p* = 0.893) demonstrated no statistically significant association with OS in the univariable Cox regression analysis.

**Figure 2:**
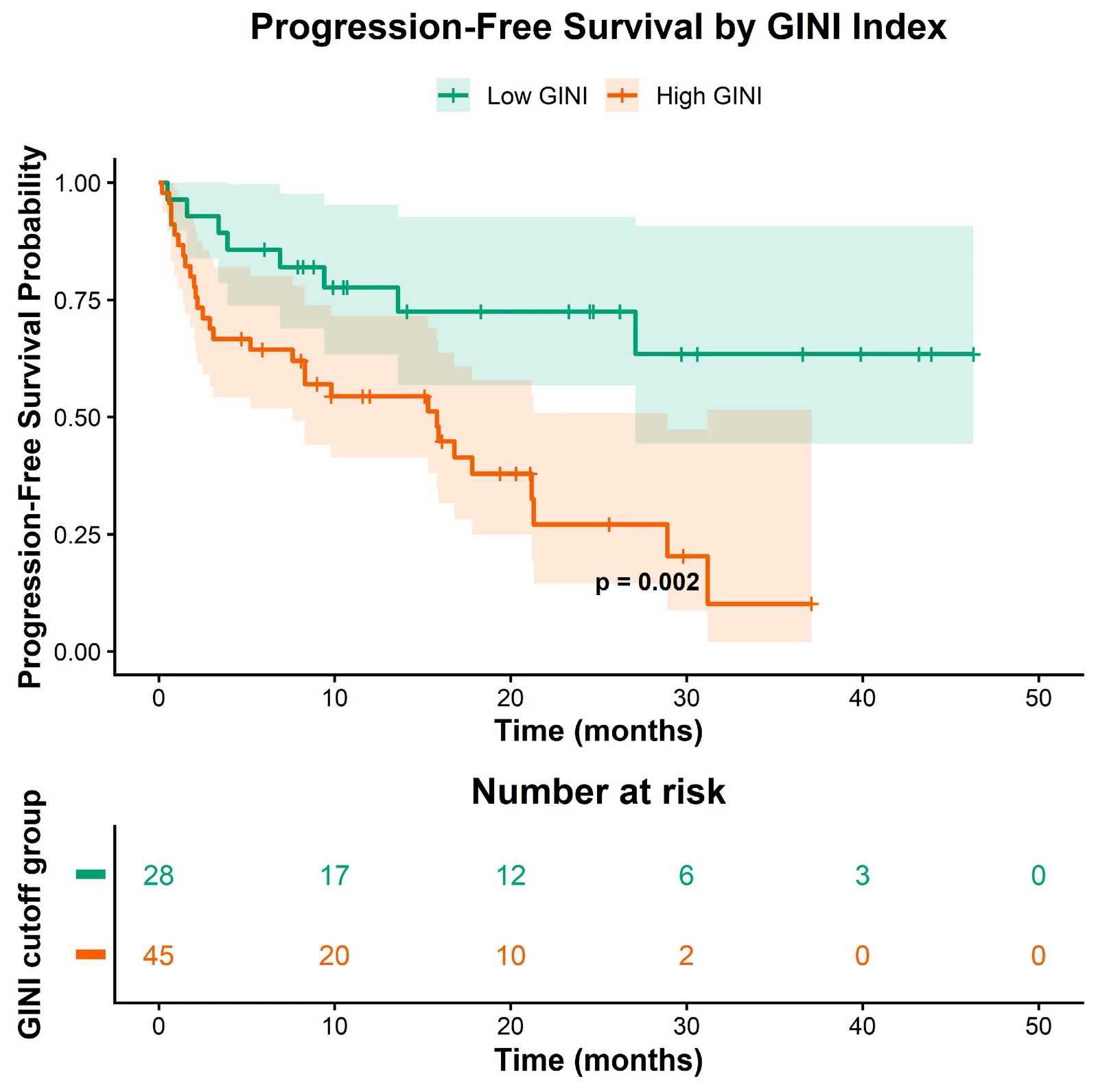
Progression-free survival KM by GINI.

For progression-free survival (PFS) [Figure 2], the high-GINI group experienced a significantly higher risk of disease progression or death compared to those in the low-GINI group adjusted HR 3.22, 95% CI 1.45–7.14, *p* = 0.004). Older age at Cemiplimab initiation was also associated with shorter PFS (adjusted HR 1.04, 95% CI 1.01–1.07, *p* = 0.011). No significant associations with PFS were found for female sex (*p* = 0.200), positive smoking history (*p* = 0.146), advanced disease stage (*p* = 0.561), surgery-based prior treatment (*p* = 0.163), or radiation-based prior treatment (*p* = 0.197) in the univariable Cox regression analysis.

### 3.4. Toxicity

The occurrence of any treatment-related toxicity did not significantly differ between the high- and low-GINI groups, showing an odds ratio (OR) of 0.8 (95% CI 0.29–2.24, *p* = 0.670). When evaluating severe toxicities, patients in the high-GINI group demonstrated a trend toward higher rates of grade ≥ 3 toxicities, with an OR of 2.83 (95% CI 0.59–9.54), though this difference did not reach statistical significance (*p* = 0.221). The most frequently reported adverse event was fatigue (26.4%), followed by pruritus (20.8%) and rash (12.5%) [Supplementary Tables S1 and S2].

### 3.5. Comparative Performance of GINI and Established Inflammatory Biomarkers

To place the prognostic performance of GINI into context, we compared its performance ability with five established inflammatory biomarkers, including the neutrophil-to-lymphocyte ratio (NLR), platelet-to-lymphocyte ratio (PLR), lymphocyte-to-monocyte ratio (LMR), prognostic nutritional index (PNI), and C-reactive protein-to-albumin ratio (CAR). Comparative analyses included 73 patients with complete biomarker and outcome data.

Receiver operating characteristic (ROC) analysis demonstrated that the PNI achieved the highest discrimination for overall mortality (AUC 0.684, 95% CI 0.558–0.811), followed by the GINI (AUC 0.659, 95% CI 0.534–0.785) and CAR (AUC 0.648, 95% CI 0.521–0.774). LMR, NLR, and PLR demonstrated lower discriminatory performance (Table 3). Pairwise comparison of ROC curves using DeLong’s test showed that the GINI significantly outperformed PLR (AUC difference 0.202, *p* = 0.003), while no significant differences were observed between the GINI and the PNI, CAR, LMR, or NLR.

After adjustment for age, sex, smoking status, disease stage, and prior definitive treatment, the CAR demonstrated the strongest association with overall survival (HR 7.25, 95% CI 1.85–28.35, *p* = 0.004), followed by the GINI (HR 3.63, 95% CI 1.41–9.36, *p* = 0.008) and the PNI (HR 2.49, 95% CI 1.05–5.91, *p* = 0.039). Similar findings were observed for progression-free survival, where the GINI (HR 4.19, 95% CI 1.78–9.82, *p* = 0.001), CAR (HR 8.24, 95% CI 2.24–30.36, *p* = 0.002), and PNI (HR 2.53, 95% CI 1.24–5.16, *p* = 0.010) remained independently associated with disease progression, whereas the NLR, PLR, and LMR were not independently associated with outcomes.

Kaplan–Meier analyses demonstrated significant differences in both overall and progression-free survival according to the ROC-derived cutoffs for the GINI, CAR, and PNI. The NLR was associated with significantly different progression-free survival but not overall survival, whereas the LMR and PLR did not significantly stratify survival outcomes. Overall, the GINI demonstrated comparable prognostic performance across multiple complementary statistical approaches.

### 3.6. Internal Validation of the Cutoff Value

The bootstrap analysis demonstrated substantial variability in the estimated GINI cutoff. The original optimal cutoff was 74.07, whereas the bootstrap median cutoff was higher at 94.35, with a wide 95% bootstrap percentile range of 9.05–528.28. The 25th and 75th percentiles were 70.33 and 101.74, respectively. The bootstrap median AUC was 0.659, with a 95% percentile range of 0.531–0.780, indicating modest discriminatory performance and considerable uncertainty. Notably, only 21.0% of bootstrap-derived cutoffs fell within ±5 GINI units of the original cutoff, further indicating substantial instability in the optimal cutoff estimate [Supplementary Table S3].

## 4. Discussion

This study evaluated the clinical utility of GINI as a prognostic biomarker for patients with cutaneous squamous cell carcinoma (cSCC) undergoing Cemiplimab therapy. Given the novelty of this multi-faceted index [8], this work represents, to the best of our knowledge, the first investigation to assess its prognostic relevance in cutaneous malignancies. Furthermore, the literature examining the specific role of the GINI score in the context of cancer immunotherapy remains exceedingly sparse, with only a single prior study addressing its prognostic value in patients receiving immune checkpoint inhibitors. Zhouxv Feng et al. [10] tested the GINI prospectively in a neoadjuvant immunochemotherapy cohort. Among 138 locally advanced esophageal SCC patients who received neoadjuvant PD-1/chemotherapy combination followed by surgery, high-GINI patients had substantially lower 3-year OS (68.7% vs. 87.8%) and DFS (63.3% vs. 82.7%), and the GINI was independently associated with poor pathological response and worse survival in multivariable analysis. Similarly in our study, we found the high-GINI group to be independently associated with inferior overall survival and progression-free survival.

Extensive effort has been dedicated to uncovering prognostic factors associated with survival outcomes for individuals undergoing ICI (immune checkpoint inhibitor) therapy. Thus far, investigations have revealed that survival heavily depends on a combination of functional metrics (performance status [22] and body mass index [23]) and classical pathological features (tumor diameter [24] and PD-L1 expression [25]). Additionally, critical genomic and cellular benchmarks have been identified, including tumor mutation burden (TMB) [26], microsatellite instability (MSI) [27], and tumor-infiltrating lymphocytes (TILs) [28]. Crucially, this ongoing search has also revealed that systemic, plasma-based inflammatory and nutritional metrics—specifically the neutrophil-to-lymphocyte ratio (NLR) [29], platelet-to-lymphocyte ratio (PLR) [30], and prognostic nutritional index (PNI) [31]—possess substantial, independent prognostic value. While the PNI relies entirely on just two blood markers (albumin for nutrition, lymphocytes for immunity), the GINI integrates six distinct parameters, capturing the interaction of nutrition, adaptive immunity, innate immunity, systemic inflammation, and pro-tumor progression factors simultaneously. By encapsulating this broader systemic spectrum, our findings suggest that the GINI score may improve prognostic risk stratification, supporting its potential as a candidate prognostic marker of worse survival outcomes and highlighting the critical impact of an amplified inflammatory state on ICI therapeutic efficacy.

The observed association between the pretreatment GINI and both overall and progression-free survival is biologically plausible, as each component of the index reflects a distinct aspect of the host inflammatory and immune response that has previously been implicated in resistance to immune checkpoint inhibition. Elevated C-reactive protein (CRP) has been associated with activation of the IL-6/STAT3 inflammatory axis, a key pathway that promotes chronic inflammation, expansion of immunosuppressive myeloid cells, and impaired cytotoxic T-cell function, ultimately reducing the efficacy of PD-1 blockade [32,33,34]. Similarly, elevated neutrophil counts are associated with increased circulating and tumor-infiltrating myeloid-derived suppressor cells (MDSCs), which suppress antitumor immunity through arginase activity, reactive oxygen species production, and inhibitory cytokine secretion [35,36]. Conversely, lymphocytes represent the principal effector cells responsible for adaptive antitumor immunity, and lymphopenia has consistently been associated with diminished responses to immune checkpoint inhibitors and inferior survival across multiple malignancies [37].

**Figure 3:**
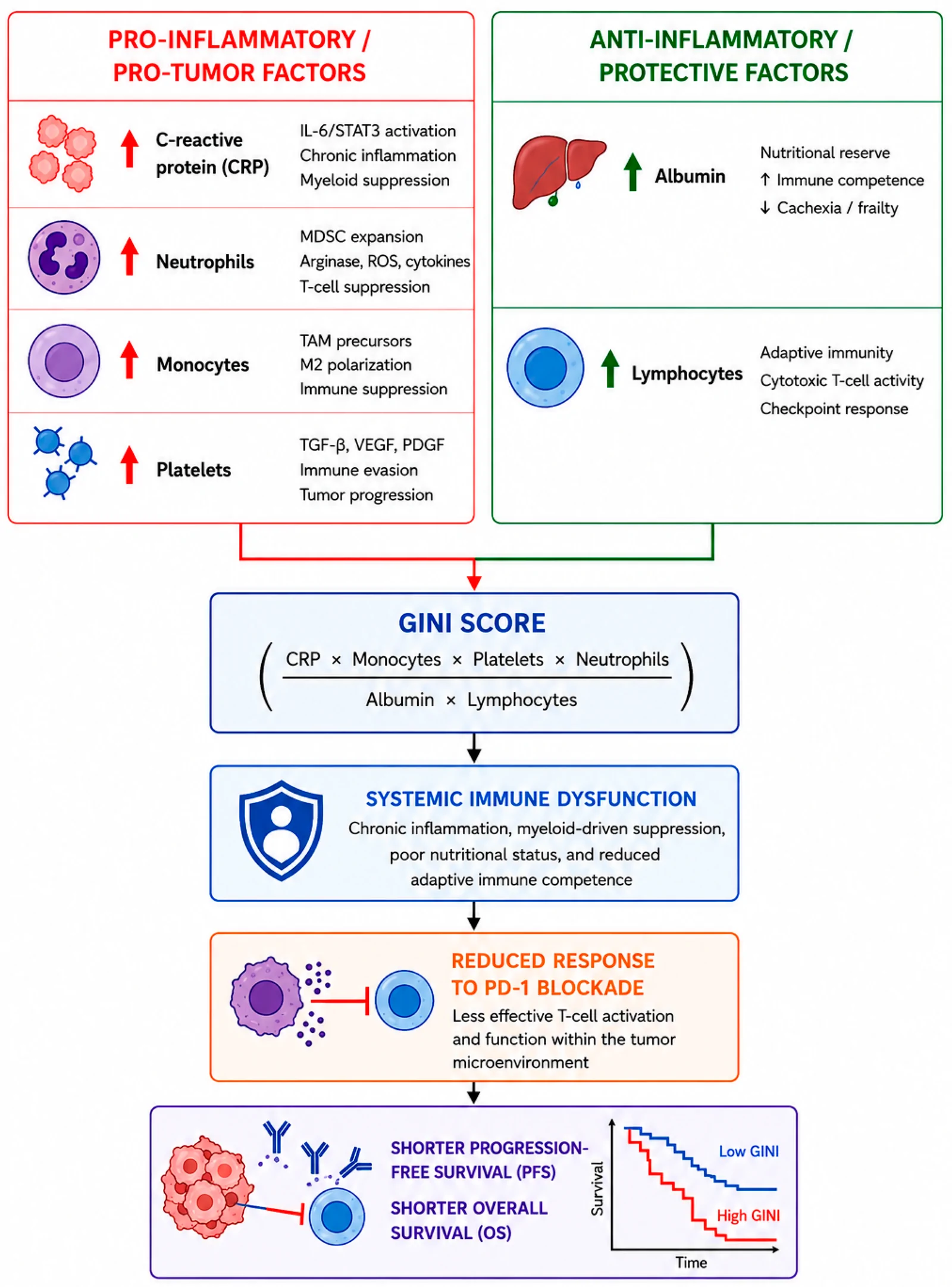
Proposed biological mechanisms linking a high GINI score with inferior outcomes during PD-1 blockade. The GINI integrates pro-inflammatory and pro-tumor factors (CRP, neutrophils, monocytes, platelets) and anti-inflammatory, protective factors (albumin, lymphocytes). A high GINI score may reflect a state of systemic inflammation, myeloid-driven immune suppression, and poor nutritional status, potentially contributing to reduced response to PD-1 blockade and shorter progression-free survival (PFS) and overall survival (OS).

Nutritional status also plays an important role in determining immunotherapy outcomes and may serve as a surrogate marker for immune status. Serum albumin is not only a marker of nutritional reserve but also reflects chronic systemic inflammation, cancer cachexia, and physiological frailty, all of which have been associated with impaired immune competence and worse oncologic outcomes [38]. Elevated platelet counts further contribute to immune evasion through the release of transforming growth factor-β (TGF-β), vascular endothelial growth factor (VEGF), and other soluble mediators that inhibit cytotoxic lymphocyte function, promote epithelial–mesenchymal transition, and facilitate metastatic dissemination [39,40]. Finally, circulating monocytes serve as precursors of tumor-associated macrophages (TAMs), one of the dominant immunosuppressive populations within the tumor microenvironment. TAMs suppress T-cell activation, promote T-cell exhaustion, and are increasingly recognized as important mediators of resistance to immune checkpoint blockade [41,42]. Taken together, these observations provide a plausible biological context for the observed association between the elevated pretreatment GINI and inferior overall and progression-free survival following Cemiplimab therapy (Figure 3). These mechanisms are proposed based on the prior literature and were not directly measured in the present study.

Cemiplimab has reshaped cSCC management across three settings. In advanced unresectable or metastatic disease, EMPOWER-CSCC-1 [43] established it as first-line standard, replacing ineffective platinum chemotherapy and EGFR inhibitors that achieved response rates of only 18% [44]. In the neoadjuvant setting, a phase 2 trial [6] reported a pathological complete response in 51% of patients with resectable stage II–IV disease—half going to surgery with no viable tumor—and many avoided planned adjuvant radiotherapy [45]. In the adjuvant setting, the phase 3 C-POST trial [7] demonstrated that Cemiplimab after surgery and radiotherapy in high-risk cSCC achieved a 2-year disease-free survival of 87% versus 64% for placebo (HR 0.32), representing a 68% reduction in recurrence or death risk. Given this multi-setting therapeutic expansion, identifying a candidate prognostic marker that can be easily calculated from routine, daily laboratory workups carries profound clinical implications. Such a tool could potentially inform risk assessment and clinical monitoring, rather than directly guide treatment selection. This is particularly critical in the neoadjuvant landscape, where initiating systemic therapy inherently delays surgical intervention—a strategy that poses a severe risk if a non-responding patient experiences disease progression, potentially rendering a primarily operable tumor inoperable. Consequently, the GINI score may offer a practical and readily available approach to prognostic risk stratification that could potentially inform treatment planning and future clinical evaluation It is important to emphasize that our findings support the GINI as a prognostic marker, whereas establishing its value as a predictive marker of treatment response would require comparison with an appropriate cohort receiving a different treatment, ideally with a formal interaction analysis between the GINI and treatment.

To our knowledge, this is the first study to directly compare the prognostic performance of GINI with multiple established inflammatory biomarkers in patients with advanced cutaneous squamous cell carcinoma treated with PD-1 inhibitors. Although several host-derived biomarkers have been proposed across different malignancies, comparative evaluations within a uniform immunotherapy-treated cohort remain limited.

Interestingly, the comparative analysis demonstrated that no single biomarker was uniformly superior across all statistical measures. While the CAR demonstrated the strongest adjusted association with both overall and progression-free survival, and the PNI achieved the highest discriminative ability on ROC analysis, the GINI demonstrated broadly comparable performance across the different analyses. This finding likely reflects the multidimensional nature of GINI, which integrates inflammatory burden, immune competence, and nutritional status into a single composite index. In contrast, traditional biomarkers such as the NLR, PLR, and LMR primarily capture isolated aspects of the systemic inflammatory response and demonstrated more limited prognostic performance in our cohort.

From a clinical perspective, these findings suggest that comprehensive host-response biomarkers may provide greater prognostic value than simple leukocyte-derived ratios in patients receiving immune checkpoint inhibitors. The efficacy of PD-1 blockade depends not only on tumor biology but also on the complex interaction between systemic inflammation, nutritional reserve, and immune function. Consequently, biomarkers incorporating multiple biological domains may more accurately reflect the host environment that influences immunotherapy response. Multidimensional host-response biomarkers integrating inflammation, nutritional status, and immune competence may better capture the biological determinants of response to PD-1 blockade than isolated leukocyte-derived ratios.

This study has several limitations. First, its retrospective, single-center design and relatively small sample size (n = 73) may have introduced selection bias and limited statistical power. Second, only 73 of the 133 patients in the original cohort had complete laboratory data and were included in the analysis, with missing CRP measurements accounting for the majority of incomplete laboratory data. As CRP is not routinely obtained in all patients, the availability of this measurement may have been non-random and potentially related to patients’ clinical status or perceived disease severity. This may have introduced selection bias, as patients with available CRP measurements could have differed systematically from those without such measurements, thereby limiting the generalizability of the findings. Third, although our findings support the potential of GINI as a prognostic marker, the specific cutoff of 74.4 showed substantial variability on bootstrap analysis and should therefore be considered exploratory and cohort-specific; validation in larger, independent cohorts is required to establish its reproducibility and clinical applicability. Fourth, the study included patients treated with Cemiplimab across neoadjuvant, adjuvant, and advanced/metastatic settings. Although this heterogeneity reflects contemporary real-world clinical practice and enhances the generalizability of our findings, differences in treatment intent and disease burden may have introduced residual confounding. Fifth, tumor response was assessed retrospectively based on radiology reports and clinical documentation rather than formal RECIST 1.1 criteria. The lack of standardized imaging protocols and systematic lesion measurements may have introduced observer variability and potential misclassification. Therefore, response-related findings should be interpreted cautiously. Finally, because of real-world logistical constraints, a small proportion of laboratory measurements were obtained shortly after treatment initiation rather than strictly before the first dose of Cemiplimab. As immune checkpoint inhibition may rapidly alter systemic inflammatory and immune parameters, these measurements may not fully represent the pretreatment physiological state.

Despite these limitations, our cohort represents a contemporary real-world population receiving Cemiplimab across its current clinical indications, supporting the clinical relevance of the findings while emphasizing the need for larger-scale prospective multicenter validation.

## 5. Conclusions

In conclusion, our study suggests that the Global Immune–Nutrition–Inflammation Index (GINI) is a candidate prognostic marker for patients with cutaneous squamous cell carcinoma (cSCC) treated with Cemiplimab. A high pretreatment GINI score was significantly associated with worse overall survival and progression-free survival across multiple treatment settings, suggesting that it may reflect compromised host immunity and heightened systemic inflammation. Given its cost-effectiveness and ready availability from routine clinical laboratory workups, the GINI score may improve prognostic risk stratification and could potentially inform risk assessment and clinical monitoring in real-world oncological practice. These findings support further investigation of GINI as a tool for patient risk stratification and future treatment evaluation strategies.

## Figures and Tables

**Table 1:** Baseline characteristics according to GINI group.

Variable	Low GINI N = 28 ^1^	High GINI N = 45 ^1^	*p*-Value
Age at Cemiplimab initiation (years)	78.84 ± 8.03	78.43 ± 13.16	0.8
Sex			>0.9
Female	5 (18%)	9 (20%)	
Male	23 (82%)	36 (80%)	
Smoking history	7 (25%)	9 (20%)	0.8
Performance status (ECOG)			0.5
ECOG ≥ 2	12 (43%)	20 (44%)	
ECOG 0	9 (32%)	9 (20%)	
ECOG 1	7 (25%)	16 (36%)	
Disease stage			0.7
Stage I	8 (29%)	11 (24%)	
Stage II	4 (14%)	12 (27%)	
Stage III	9 (32%)	13 (29%)	
Stage IV	7 (25%)	9 (20%)	
T stage			>0.9
T1	7 (25%)	12 (27%)	
T2	7 (25%)	12 (27%)	
T3	7 (25%)	12 (27%)	
T4	3 (11%)	2 (4.4%)	
Tx	4 (14%)	7 (16%)	
N stage			>0.9
N0	22 (79%)	37 (82%)	
N1	1 (3.6%)	2 (4.4%)	
N2	1 (3.6%)	2 (4.4%)	
N2a	1 (3.6%)	0 (0%)	
N2b	1 (3.6%)	1 (2.2%)	
N2c	1 (3.6%)	1 (2.2%)	
N3	0 (0%)	1 (2.2%)	
N3b	1 (3.6%)	1 (2.2%)	
M stage			>0.9
M0	27 (96%)	43 (96%)	
M1	1 (3.6%)	2 (4.4%)	
Primary tumor location			0.6
Forearm	0 (0%)	1 (2.2%)	
Hands	1 (3.6%)	1 (2.2%)	
Head and neck	20 (71%)	27 (60%)	
Lower limb	1 (3.6%)	4 (8.9%)	
Shoulder	0 (0%)	3 (6.7%)	
Trunk	0 (0%)	2 (4.4%)	
Unknown primary	6 (21%)	7 (16%)	
Any immunosuppression	6 (21%)	17 (38%)	0.2
Prior definitive treatment			0.6
Chemoradiotherapy	2 (7.1%)	0 (0%)	
None	9 (32%)	11 (24%)	
Radiotherapy	3 (11%)	6 (13%)	
Surgery	4 (14%)	7 (16%)	
Surgery + adjuvant CRT	2 (7.1%)	5 (11%)	
Surgery + adjuvant RT	8 (29%)	16 (36%)	
Albumin (g/dL)	4.10 (3.90, 4.40)	3.70 (3.38, 4.10)	<0.001
C-reactive protein (CRP)	0.25 (0.13, 0.56)	2.86 (1.44, 5.57)	<0.001
Neutrophils	4.30 (3.25, 5.25)	6.30 (4.70, 8.60)	<0.001
Lymphocytes	1.75 (1.05, 2.40)	1.20 (0.90, 1.90)	0.10
Monocytes	0.63 (0.45, 0.83)	0.70 (0.50, 0.83)	0.7
Platelets	179.50 (144.50, 203.00)	241.00 (189.00, 300.00)	<0.001

^1^ Continuous variables are presented as median and interquartile range; categorical variables are presented as n (%).

**Table 2:** Response to treatment by GINI group.

Variable	Low GINI N = 28	High GINI N = 45	*p*-Value
PD-1 treatment period	9.25 (3.70, 19.10)	5.80 (2.10, 12.10)	0.066
Time to best response (months)	5.95 (2.55, 8.90)	3.90 (2.00, 7.90)	0.3
Best response			0.7
Complete Response	11 (42%)	10 (28%)	
Partial Response	11 (42%)	18 (50%)	
Progressive Disease	3 (12%)	5 (14%)	
Stable Disease	1 (4%)	3 (8.3%)	
Death	7 (25%)	26 (58%)	0.008

**Table 3:** Comparative prognostic performance of inflammatory biomarkers for overall survival and progression-free survival.

Biomarker	AUC (95% CI)	Adjusted OS HR (95% CI)	Adjusted PFS HR (95% CI)	OS Log-Rank *p*	PFS Log-Rank *p*
GINI	0.659 (0.534–0.785)	3.63 (1.41–9.36)	4.19 (1.78–9.82)	0.008	0.002
CAR	0.648 (0.521–0.774)	7.25 (1.85–28.35)	8.24 (2.24–30.36)	0.013	0.005
PNI	0.684 (0.558–0.811)	2.49 (1.05–5.91)	2.53 (1.24–5.16)	<0.001	0.001
NLR	0.534 (0.397–0.671)	2.07 (0.75–5.71)	2.51 (0.96–6.56)	0.063	0.022
LMR	0.567 (0.433–0.702)	1.96 (0.78–4.94)	2.08 (0.86–5.02)	0.102	0.169
PLR	0.458 (0.323–0.593)	0.97 (0.47–2.00)	1.33 (0.67–2.64)	0.832	0.493

## Data Availability

The raw data supporting the conclusions of this article will be made available by the authors upon request.

## Supplementary Materials

The following supporting information can be downloaded at: https://www.mdpi.com/article/10.3390/cancers18172862/s1, Figure S1: Inclusion and exclusion flow chart; Table S1: Specific Adverse Events in the Final GINI Cohort; Table S2: Specific Adverse Events by GINI Group. Table S3: ROC and Bootstrap Validation.

## Abbreviations

The following abbreviations are used in this manuscript:

GINI	Global Immune–Nutrition–Inflammation Index
cSCC	Cutaneous Squamous Cell Carcinoma
NLR	Neutrophil-to-Lymphocyte Ratio
PNI	Prognostic Nutritional Index
LMR	Lymphocyte-to-Monocyte Ratio
PLR	Platelet-to-Lymphocyte Ratio
CAR	C-reactive Protein-to-Albumin Ratio

## References

[1] Rembielak A., Ajithkumar T. (2019). Non-Melanoma Skin Cancer—An Underestimated Global Health Threat?. Clin. Oncol..

[2] Zargham H., Strasswimmer J. (2022). Rapid response to cemiplimab for advanced cutaneous squamous cell carcinoma. JAAD Case Rep..

[3] Dessinioti C., Pitoulias M., Stratigos A.J. (2022). Epidemiology of advanced cutaneous squamous cell carcinoma. J. Eur. Acad. Dermatol. Venereol..

[4] Migden M.R., Rischin D., Schmults C.D., Guminski A., Hauschild A., Lewis K.D., Chung C.H., Hernandez-Aya L.F., Lim A.M., Chang A.L.S. (2018). PD-1 Blockade with Cemiplimab in Advanced Cutaneous Squamous-Cell Carcinoma. N. Engl. J. Med..

[5] Migden M.R., Khushalani N.I., Chang A.L.S., Lewis K.D., Schmults C.D., Hernandez-Aya L., Meier F., Schadendorf D., Guminski A., Hauschild A. (2020). Cemiplimab in locally advanced cutaneous squamous cell carcinoma: Results from an open-label, phase 2, single-arm trial. Lancet Oncol..

[6] Gross N.D., Miller D.M., Khushalani N.I., Divi V., Ruiz E.S., Lipson E.J., Meier F., Su Y.B., Swiecicki P.L., Atlas J. (2022). Neoadjuvant Cemiplimab for Stage II to IV Cutaneous Squamous-Cell Carcinoma. N. Engl. J. Med..

[7] Rischin D., Porceddu S., Day F., Brungs D.P., Christie H., Jackson J.E., Stein B.N., Su Y.B., Ladwa R., Adams G. (2025). Adjuvant Cemiplimab or Placebo in High-Risk Cutaneous Squamous-Cell Carcinoma. N. Engl. J. Med..

[8] Topkan E., Selek U., Pehlivan B., Kucuk A., Ozturk D., Ozdemir B.S., Besen A.A., Mertsoylu H. (2023). The Prognostic Value of the Novel Global Immune-Nutrition-Inflammation Index (GINI) in Stage IIIC Non-Small Cell Lung Cancer Patients Treated with Concurrent Chemoradiotherapy. Cancers.

[9] Aoyama T., Hashimoto I., Maezawa Y., Hara K., Yamamoto S., Esashi R., Tamagawa A., Cho H., Tanabe M., Morita J. (2024). Global Immune-Nutrition-Information Index Is Independent Prognostic Factor for Gastric Cancer Patients Who Received Curative Treatment. Cancer Diagn. Progn..

[10] Feng Z., Yi H., Xiao J., Huang Z., Tu Y., Liu B. (2025). The global immune-nutrition-inflammation index predicts pathological response and survival in esophageal squamous cell carcinoma treated with neoadjuvant immunochemotherapy. Front. Nutr..

[11] Bozkurt O., Gönül R., Kaya B.U., Zararsiz G.E., İnanc M., Özkan M. (2025). The prognostic importance of the global immune-nutrition-information index (GINI) in patients with Ras wild type metastatic colorectal cancer. Sci. Rep..

[12] Aydin A.A., Yuceer R.O. (2024). Unraveling the Predictive Value of the Novel Global Immune-Nutrition-Inflammation Index (GINI) on Survival Outcomes in Patients with Grade 4 Adult-Type Diffuse Gliomas. Curr. Oncol..

[13] Seretis K., Sfaelos K., Boptsi E., Gaitanis G., Bassukas I.D. (2024). The Neutrophil-to-Lymphocyte Ratio as a Biomarker in Cutaneous Oncology: A Systematic Review of Evidence beyond Malignant Melanoma. Cancers.

[14] Kartolo A., Holstead R., Khalid S., Emack J., Hopman W., Robinson A., Baetz T. (2020). Serum neutrophil-to-lymphocyte ratio and platelet-to-lymphocyte ratio in prognosticating immunotherapy efficacy. Immunotherapy.

[15] Gambichler T., Brune J., Rüth J., Abu Rached N., Boms S., Weyer-Fahlbusch S.S., Kreuter A., Hyun J., Becker J.C., Susok L. (2026). Predictors of response and survival in cemiplimab–treated cutaneous squamous cell carcinoma: Multicenter real-world evidence from Germany. J. Cancer Res. Clin. Oncol..

[16] Wang Y., Wang J., Xiao B., Wang Y., Huang F., Jiang Y., Liu T. (2025). Prognostic significance of prognostic nutritional index in patients with head and neck squamous cell carcinoma. Front. Immunol..

[17] Dai M., Wu W. (2023). Prognostic role of C-reactive protein to albumin ratio in cancer patients treated with immune checkpoint inhibitors: A meta-analysis. Front. Oncol..

[18] Oken M.M., Creech R.H., Davis T.E. (1982). Toxicology and response criteria of the Eastern Cooperative Oncology Group. Am. J. Clin. Oncol..

[19] Amin M.B., Greene F.L., Edge S.B., Compton C.C., Gershenwald J.E., Brookland R.K., Meyer L., Gress D.M., Byrd D.R., Winchester D.P. (2017). The Eighth Edition AJCC Cancer Staging Manual: Continuing to build a bridge from a population-based to a more ‘personalized’ approach to cancer staging. CA Cancer J. Clin..

[20] Eisenhauer E.A., Therasse P., Bogaerts J., Schwartz L.H., Sargent D., Ford R., Dancey J., Arbuck S., Gwyther S., Mooney M. (2009). New response evaluation criteria in solid tumours: Revised RECIST guideline (version 1.1). Eur. J. Cancer.

[21] National Cancer Institute (2017). Common Terminology Criteria for Adverse Events (CTCAE) Common Terminology Criteria for Adverse Events (CTCAE) v5.0. https://www.meddra.org/.

[22] Ge W., Wu N., Chen C.-I., Inocencio T.J., LaFontaine P.R., Seebach F., Fury M., Harnett J., Ruiz E.S. (2024). Real-World Treatment Patterns and Outcomes of Cemiplimab in Patients with Advanced Cutaneous Squamous Cell Carcinoma Treated in US Oncology Practices. Cancer Manag. Res..

[23] An Y., Wu Z., Wang N., Yang Z., Li Y., Xu B., Sun M. (2020). Association between body mass index and survival outcomes for cancer patients treated with immune checkpoint inhibitors: A systematic review and meta-analysis. J. Transl. Med..

[24] Hakozaki T., Hosomi Y., Kitadai R., Kitagawa S., Okuma Y. (2020). Efficacy of immune checkpoint inhibitor monotherapy for patients with massive non-small-cell lung cancer. J. Cancer Res. Clin. Oncol..

[25] Kilickap S., Baramidze A., Sezer A., Özgüroğlu M., Gumus M., Bondarenko I., Gogishvili M., Nechaeva M., Schenker M., Cicin I. (2025). Cemiplimab Monotherapy for First-Line Treatment of Patients with Advanced NSCLC with PD-L1 Expression of 50% or Higher: Five-Year Outcomes of EMPOWER-Lung 1. J. Thorac. Oncol..

[26] Goodman A.M., Kato S., Bazhenova L., Patel S.P., Frampton G.M., Miller V., Stephens P.J., Daniels G.A., Kurzrock R. (2017). Tumor mutational burden as an independent predictor of response to immunotherapy in diverse cancers. Mol. Cancer Ther..

[27] Petrelli F., Ghidini M., Ghidini A., Tomasello G. (2020). Outcomes following Immune Checkpoint Inhibitor Treatment of Patients with Microsatellite Instability-High Cancers: A Systematic Review and Meta-analysis. JAMA Oncol..

[28] Zou Y., Zou X., Zheng S., Tang H., Zhang L., Liu P., Xie X. (2020). Efficacy and predictive factors of immune checkpoint inhibitors in metastatic breast cancer: A systematic review and meta-analysis. Ther. Adv. Med. Oncol..

[29] Jiang T., Qiao M., Zhao C., Li X., Gao G., Su C., Ren S., Zhou C. (2018). Pretreatment neutrophil-to-lymphocyte ratio is associated with outcome of advanced-stage cancer patients treated with immunotherapy: A meta-analysis. Cancer Immunol. Immunother..

[30] Xu H., He A., Liu A., Tong W.X., Cao D. (2019). Evaluation of the prognostic role of platelet-lymphocyte ratio in cancer patients treated with immune checkpoint inhibitors: A systematic review and meta-analysis. Int. Immunopharmacol..

[31] Ni L., Huang J., Ding J., Kou J., Shao T., Li J., Gao L., Zheng W., Wu Z. (2022). Prognostic Nutritional Index Predicts Response and Prognosis in Cancer Patients Treated with Immune Checkpoint Inhibitors: A Systematic Review and Meta-Analysis. Front. Nutr..

[32] Johnson D.E., O’Keefe R.A., Grandis J.R. (2018). Targeting the IL-6/JAK/STAT3 signalling axis in cancer. Nat. Rev. Clin. Oncol..

[33] Yu H., Pardoll D., Jove R. (2009). STATs in cancer inflammation and immunity: A leading role for STAT3. Nat. Rev. Cancer.

[34] Hailemichael Y., Johnson D.H., Abdel-Wahab N., Foo W.C., Bentebibel S.-E., Daher M., Haymaker C., Wani K., Saberian C., Ogata D. (2022). Interleukin-6 blockade abrogates immunotherapy toxicity and promotes tumor immunity. Cancer Cell.

[35] Veglia F., Sanseviero E., Gabrilovich D.I. (2021). Myeloid-derived suppressor cells in the era of increasing myeloid cell diversity. Nat. Rev. Immunol..

[36] Gabrilovich D.I. (2017). Myeloid-derived suppressor cells. Cancer Immunol. Res..

[37] Farber D.L., Yudanin N.A., Restifo N.P. (2014). Human memory T cells: Generation, compartmentalization and homeostasis. Nat. Rev. Immunol..

[38] Baracos V.E., Martin L., Korc M., Guttridge D.C., Fearon K.C.H. (2018). Cancer-associated cachexia. Nat. Rev. Dis. Prim..

[39] Schlesinger M. (2018). Role of platelets and platelet receptors in cancer metastasis. J. Hematol. Oncol..

[40] Gay L.J., Felding-Habermann B. (2011). Contribution of platelets to tumour metastasis. Nat. Rev. Cancer..

[41] Mantovani A., Marchesi F., Malesci A., Laghi L., Allavena P. (2017). Tumour-associated macrophages as treatment targets in oncology. Nat. Rev. Clin. Oncol..

[42] Cassetta L., Pollard J.W. (2018). Targeting macrophages: Therapeutic approaches in cancer. Nat. Nat. Rev. Drug Discov..

[43] Hughes B.G.M., Guminski A., Bowyer S., Migden M.R., Schmults C.D., Khushalani N.I., Chang A.L.S., Grob J.-J., Lewis K.D., Ansstas G. (2025). A phase 2 open-label study of cemiplimab in patients with advanced cutaneous squamous cell carcinoma (EMPOWER-CSCC-1): Final long-term analysis of groups 1, 2, and 3, and primary analysis of fixed-dose treatment group 6. J. Am. Acad. Dermatol..

[44] Cowey C.L., Robert N.J., Espirito J.L., Davies K., Frytak J., Lowy I., Fury M.G. (2020). Clinical outcomes among unresectable, locally advanced, and metastatic cutaneous squamous cell carcinoma patients treated with systemic therapy. Cancer Med..

[45] Ferrarotto R., Amit M., Nagarajan P., Rubin M.L., Yuan Y., Bell D., El-Naggar A.K., Johnson J.M., Morrison W.H., Rosenthal D.I. (2021). Pilot phase II trial of neoadjuvant immunotherapy in locoregionally advanced, resectable cutaneous squamous cell carcinoma of the head and neck. Clin. Cancer Res..

