# Supplementary material for: The Global Immune–Nutrition–Inflammation Index (GINI) as a Candidate Prognostic Marker in Patients with Cutaneous Squamous Cell Carcinoma Treated with Cemiplimab
Source: Cancers (Basel). 2026 Sep 4;18(17):2862. doi: 10.3390/cancers18172862 (PMC13565106; doi:10.3390/cancers18172862)
Supplement: Supplementary file 1 [file cancers-18-02862-s001.zip › Figure S1.pdf]

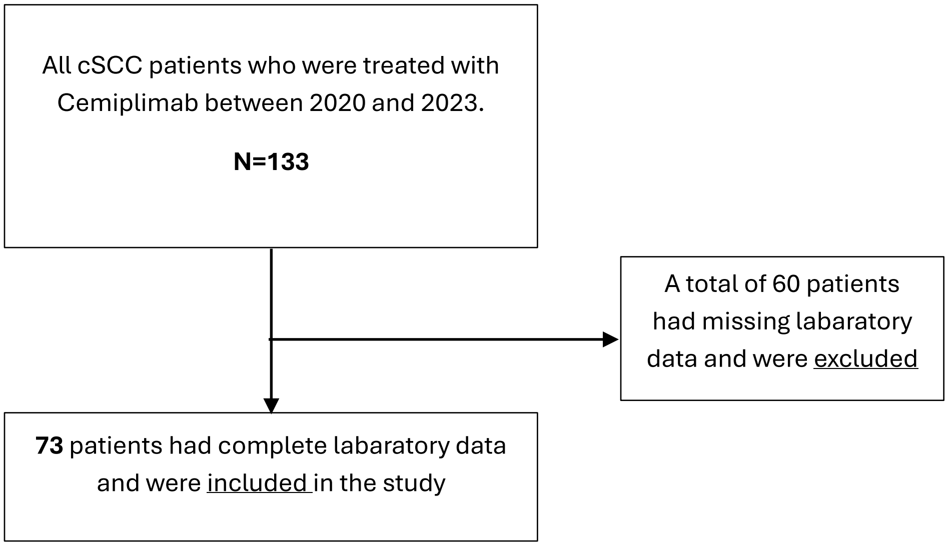

Figure S1. Inclusion and exclusion flow chart
