# Supplementary material for: The Global Immune–Nutrition–Inflammation Index (GINI) as a Candidate Prognostic Marker in Patients with Cutaneous Squamous Cell Carcinoma Treated with Cemiplimab
Source: Cancers (Basel). 2026 Sep 4;18(17):2862. doi: 10.3390/cancers18172862 (PMC13565106; doi:10.3390/cancers18172862)
Supplement: Supplementary file 1 [file cancers-18-02862-s001.zip › supplemntal Table S1 and S2.pdf]

Table S1. Specific Adverse Events in the Final GINI Cohort

Adverse Event	Frequency
Fatigue	19 (26.4%)
Pruritus	15 (20.8%)
Rash	9 (12.5%)
Other	8 (11.1%)
Diarrhea	7 (9.7%)
Hypothyroidism	6 (8.3%)
Pneumonitis	6 (8.3%)
Weakness	6 (8.3%)
Dermatitis	4 (5.6%)
Nausea	4 (5.6%)
Adrenal insufficiency	2 (2.8%)
Arthralgia	2 (2.8%)
Arthritis	2 (2.8%)
Cytopenia	2 (2.8%)
Hepatitis	2 (2.8%)
Ageusia	1 (1.4%)
Colitis	1 (1.4%)
Fever	1 (1.4%)
Guillain-Barré syndrome	1 (1.4%)
Hyperthyroidism	1 (1.4%)
Myasthenia gravis	1 (1.4%)
Myasthenia–myocarditis–myositis	1 (1.4%)
Myocarditis	1 (1.4%)
Nephritis	1 (1.4%)
Neuropathy	1 (1.4%)
Raynaud's phenomenon	1 (1.4%)
Weight loss	1 (1.4%)
Anemia	0 (0.0%)
Asthenia	0 (0.0%)
Infusion reactions	0 (0.0%)
Psoriasis	0 (0.0%)
Type 1 diabetes mellitus	0 (0.0%)

Table S2. Specific Adverse Events by GINI Group

Variable	Low GINI	High GINI
Adrenal insufficiency	1 (3.7%)	1 (2.2%)
Ageusia	0 (0.0%)	1 (2.2%)
Anemia	0 (0.0%)	0 (0.0%)
Arthralgia	1 (3.7%)	1 (2.2%)
Arthritis	0 (0.0%)	2 (4.4%)
Asthenia	0 (0.0%)	0 (0.0%)
Colitis	0 (0.0%)	1 (2.2%)
Cytopenia	1 (3.7%)	1 (2.2%)
Dermatitis	2 (7.4%)	2 (4.4%)
Diarrhea	3 (11.1%)	4 (8.9%)
Fatigue	10 (37.0%)	9 (20.0%)
Fever	1 (3.7%)	0 (0.0%)
Guillain-Barré syndrome	1 (3.7%)	0 (0.0%)
Hepatitis	1 (3.7%)	1 (2.2%)
Hyperthyroidism	0 (0.0%)	1 (2.2%)
Hypothyroidism	2 (7.4%)	4 (8.9%)
Infusion reactions	0 (0.0%)	0 (0.0%)
Myasthenia gravis	0 (0.0%)	1 (2.2%)
Myasthenia–myocarditis–myositis	1 (3.7%)	0 (0.0%)
Myocarditis	0 (0.0%)	1 (2.2%)
Nausea	3 (11.1%)	1 (2.2%)
Nephritis	1 (3.7%)	0 (0.0%)
Neuropathy	0 (0.0%)	1 (2.2%)
Other	2 (7.4%)	6 (13.3%)
Pneumonitis	2 (7.4%)	4 (8.9%)
Pruritus	4 (14.8%)	11 (24.4%)
Psoriasis	0 (0.0%)	0 (0.0%)
Rash	2 (7.4%)	7 (15.6%)
Raynaud's phenomenon	0 (0.0%)	1 (2.2%)
Type 1 diabetes mellitus	0 (0.0%)	0 (0.0%)
Weakness	1 (3.7%)	5 (11.1%)
Weight loss	0 (0.0%)	1 (2.2%)
