# Supplementary material for: The Global Immune–Nutrition–Inflammation Index (GINI) as a Candidate Prognostic Marker in Patients with Cutaneous Squamous Cell Carcinoma Treated with Cemiplimab
Source: Cancers (Basel). 2026 Sep 4;18(17):2862. doi: 10.3390/cancers18172862 (PMC13565106; doi:10.3390/cancers18172862)
Supplement: Supplementary file 1 [file cancers-18-02862-s001.zip › supplemntal Table S3.pdf]

Table S3. ROC and Bootstrap Validation

Measure	Value
Original cutoff	74.0707294
Bootstrap median cutoff	94.3495192
Bootstrap cutoff 2.5th percentile	9.0491667
Bootstrap cutoff 25th percentile	70.3337862
Bootstrap cutoff 75th percentile	101.7437362
Bootstrap cutoff 97.5th percentile	528.2827456
Bootstrap median AUC	0.6592988
Bootstrap AUC 2.5th percentile	0.5309166
Bootstrap AUC 97.5th percentile	0.7798450
Proportion of bootstrap cutoffs within ±5 GINI units of original	0.2101000
